# Genetic targeting of Purkinje fibres by *Sema3a-CreERT2*

**DOI:** 10.1038/s41598-018-20829-9

**Published:** 2018-02-05

**Authors:** Yan Li, Xueying Tian, Huan Zhao, Lingjuan He, Shaohua Zhang, Xiuzhen Huang, Hui Zhang, Lucile Miquerol, Bin Zhou

**Affiliations:** 1 The State Key Laboratory of Cell Biology, CAS Center for Excellence in Molecular Cell Science, Shanghai Institute of Biochemistry and Cell Biology, Chinese Academy of Sciences, University of Chinese Academy of Sciences, Shanghai, 200031 China; 2 Key Laboratory of Nutrition and Metabolism, Institute for Nutritional Sciences, Shanghai Institutes for Biological Sciences, Chinese Academy of Sciences, University of Chinese Academy of Sciences, Shanghai, 200031 China; 3 Aix Marseille University, CNRS, IBDM UMR 7288, 13288 Marseille, France; 4 Key Laboratory of Regenerative Medicine of the Ministry of Education, Institute of Aging and Regenerative Medicine, Jinan University, Guangzhou, 510632 China; 5 School of Life Science and Technology, ShanghaiTech University, Shanghai, 201210 China

## Abstract

The maintenance of the heart rhythm and the conduction of excitatory signals require changing excitatory signals via electrical activity and coordination by communication between working and conductive cardiomyocytes. Understanding how the ventricular conduction system is established provides novel insights into the pathophysiological progress of cardiac arrhythmias. However, the major hurdle in this field is the lack of a specific genetic tool that targets the Purkinje fibres of the ventricular conduction system and no other types of cardiomyocytes or coronary vessels. Here, we generated a *Sema3a-CreERT2* knock-in mouse line to test its specificity for genetically labelled Purkinje fibres. We found that Sema3a was expressed in the subendocardial layer of the trabecular myocardium in the embryonic heart and was restricted to the Purkinje fibres in the adult heart. A fate mapping study based on the *Sema3a-CreERT2* line revealed that the Sema3a^+^ cardiomyocytes were restricted to the fate of Purkinje fibres in the perinatal but not the embryonic stage. Collectively, our study provides a new genetic tool, i.e., *Sema3a-CreERT2*, for studying the molecular mechanisms that regulate the function of Purkinje fibres.

## Introduction

The cardiac conduction system controls cardiac contraction by regulating the propagation of the electrical activity through the heart. Defects in the cardiac conduction system may lead to cardiac arrhythmias. Cardiac contraction begins in the right atrium, where it is initiated by the sinoatrial node (SA node). Then, the electrical activity is delayed at the atrioventricular node (AV node) and subsequently rapidly propagated throughout the ventricular wall through the fast-conducting atrioventricular bundle (the bundle of His and the bundle branches), which is localized at the crest of interventricular septum, and the peripheral left and right ventricular Purkinje fibres that are localized to the subendocardial surface of the myocardial wall^1^. The Purkinje fibres ensure the rapid propagation of electric pulses that permit the efficient and effective pumping of blood from the ventricles. Understanding the molecular mechanisms that regulate Purkinje fibre development, formation, maintenance and proper function will provide insights into the pathophysiological process of cardiac arrhythmias. A recent study demonstrated that *Hcn4-CreERT2* labels the conduction system including the sinoatrial node, a subset of the atrioventricular node, the bundle of His, the bundle branches and the Purkinje fibres^2,3^. Additionally, *Hcn4-CreERT2* also labels a subset of endothelial cells in the developing heart^2,3^. *Cntn2-Cre* is another genetic tool for the cardiac conduction system that specifically targets the SA node, AV node, left and right bundle branches and the Purkinje fibres^4^. Previous research has revealed that connexin 40 (Cx40) is largely restricted to the ventricular conduction system (VCS), and it is also expressed in atrial cardiomyocytes and coronary arterial endothelial cells^5,6^. The genetic tool *Cx40-CreERT2* targets both atrial and VCS cardiomyocytes, as well as coronary endothelial cells^1,7^. Therefore, the generation of a new tool that specifically targets the Purkinje fibres without targeting other cells may be valuable for specifically studying the function of Purkinje fibres.

Sema3a is a member of the semaphorin family that is conserved from invertebrates to vertebrates. Recent studies indicate that Sema3a has an important function in the development of the nervous system^8,9^. Sema3a could be a chemorepellent that inhibits the outgrowth of axons, especially peripheral axons, to maintain a normal neuronal pattern^10^. The overexpression of Sema3a can lead to nerve diseases, such as schizophrenia, but defects in Sema3a result in abnormal neuronal innervation^11,12^. Moreover, Sema3a also plays physiological roles in immunoregulation^13^ and cancer development^14^. A recent study indicates that Sema3a can maintain a normal heart rhythm by mediating the patterning of the sympathetic innervation^15^. Interestingly, the expression of Sema3a is detected in the Purkinje fibres and a subset of trabecular cardiomyocytes, but to our knowledge, lineage tracing results regarding Sema3a in the cardiac conduction system have not yet been reported. In our study, we delineated the expression patterns of Sema3a in the developing and adult heart. The genetic lineage tracing analysis with *Sema3a-CreERT2* revealed that Sema3a specifically labels Purkinje fibres in the adult heart, and the fate of these specialized cardiomyocytes is actually determined in the perinatal stage. These results expand our knowledge about the development of the VCS and provide a new genetic mouse tool for the study of Purkinje fibre function.

## Results

### Generation and characterization of the *Sema3a-CreERT2* mouse line

**Figure 1:**
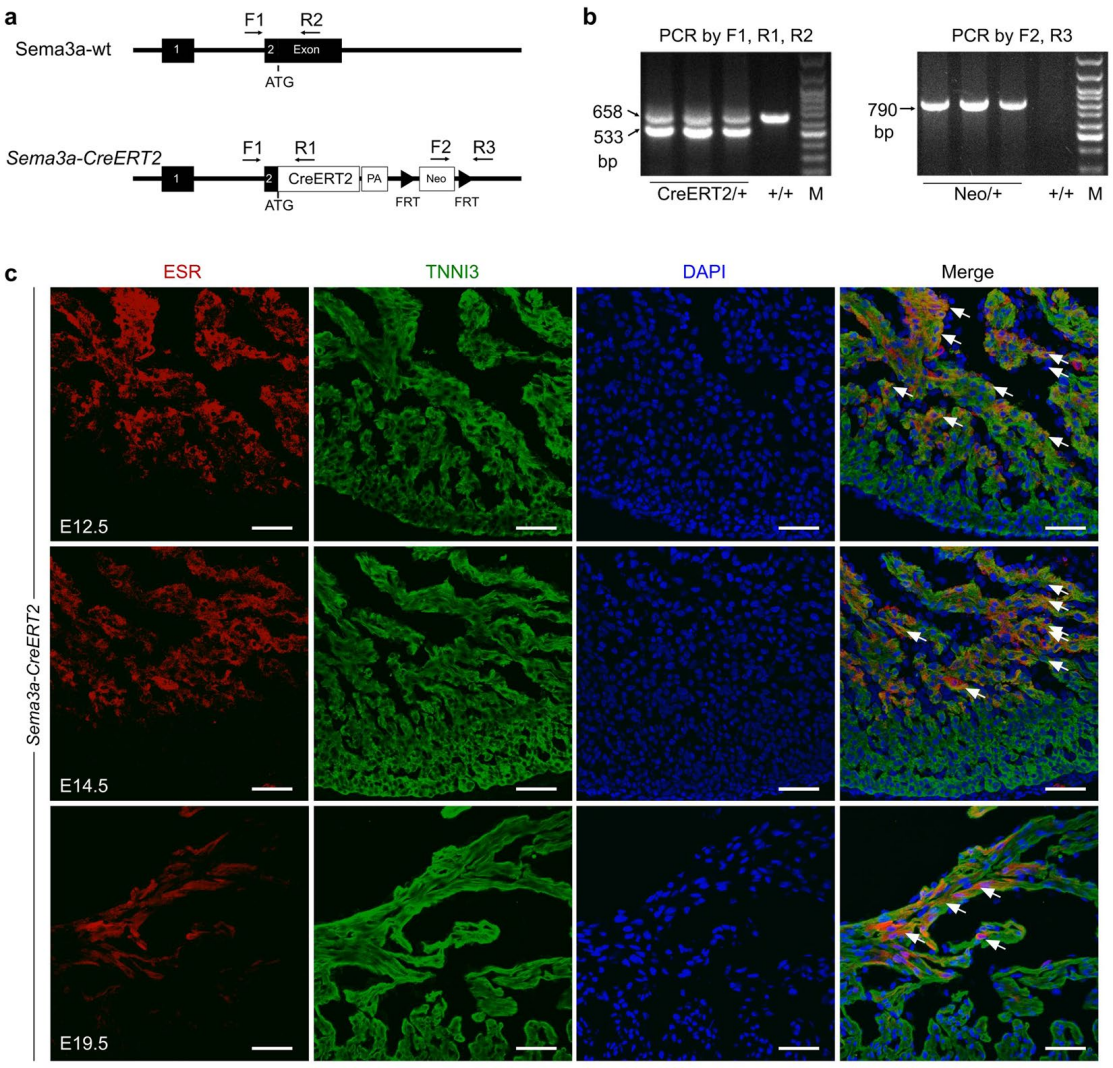
Generation and characterization of the *Sema3a-CreERT2* knock-in mouse line. (**a**) Schematic diagrams showing the wild-type (wt) allele and the CreERT2 knock-in Sema3a allele. The arrows indicate the primers that were designed for genotyping. (**b**) Genotyping of the *Sema3a-CreERT2* allele by PCR. The band size is presented on the left. M, molecular marker. (**c**) Immunostaining for oestrogen receptor (ESR) as a surrogate for Sema3a on E12.5, E14.5 and E19.5 *Sema3a-CreERT2* heart sections. The arrows indicate Sema3a^+^ cardiomyocytes. Scale bars, 100 µm.

We generated a *Sema3a-CreERT2* knock-in mouse line by homologous recombination using a conventional ES cell-based targeting strategy. These *Sema3a-CreERT2* mice expressed the tamoxifen-inducible CreERT2 recombinase (CreER^T2^) under the endogenous transcriptional control of the Sema3a gene (Fig. 1a). DNA from the tails of heterozygous mice (*Sema3a-CreERT2*) and wild-type littermates (*Sema3a*^+/+^) was extracted for polymerase chain reaction (PCR) experiments. The *Sema3a-CreERT2* mice had two bands: one band corresponded to the mutant knock-in allele, and the other band corresponded to the wild-type allele (Fig. 1b). Un-cropped PCR gel pictures are presented in Supplementary Fig. 1. Homozygous mutant mice were not obtained in our study due to the high postnatal mortality of Sema3a null mutations^11^. To detect the function of these heterozygous mice, we tested the ejection fraction and the fractional shortening of 8-week-old mice and found no significant difference between the *Sema3a-CreERT2* and the *Sema3a*^+/+^ littermate control mice (Supplementary Fig. 2a,b). ECG and echocardiography revealed that the *Sema3a-CreERT2* mice exhibited normal heart function at the age of 8 weeks (Supplementary Fig. 2c). To test whether the knock-in allele was expressed as expected, we first used oestrogen receptor (ESR) antibody staining as a surrogate for Sema3a expression in embryonic lung and bone tissue. ESR/Sema3a was highly expressed in both organs (Supplementary Fig. 3), which is consistent with a previous study^16^. Then, we co-stained for ESR and TNNI3 in E12.5, E14.5 and E19.5 embryonic heart sections. The immunostaining results revealed that ESR/Sema3a expression was largely restricted to the trabecular myocardium, but minimal staining was detected in the compact layer in the E12.5 and E14.5 embryonic hearts (Fig. 1c). At E19.5, ESR/Sema3a was expressed in a subset of subendocardial trabecular cardiomyocytes that were located along the side of the ventricular chamber (Fig. 1c). ESR/Sema3a was not detected in the cardiomyocytes or the coronary arteries of the compact myocardium (Fig. 1c), which is consistent with a previous study of Sema3a expression^15^. Taken together, these data demonstrated the successful generation of the *Sema3a-CreERT2* mouse line.

### *Sema3a-CreERT2* labels Purkinje fibres in the adult mouse heart

**Figure 2:**
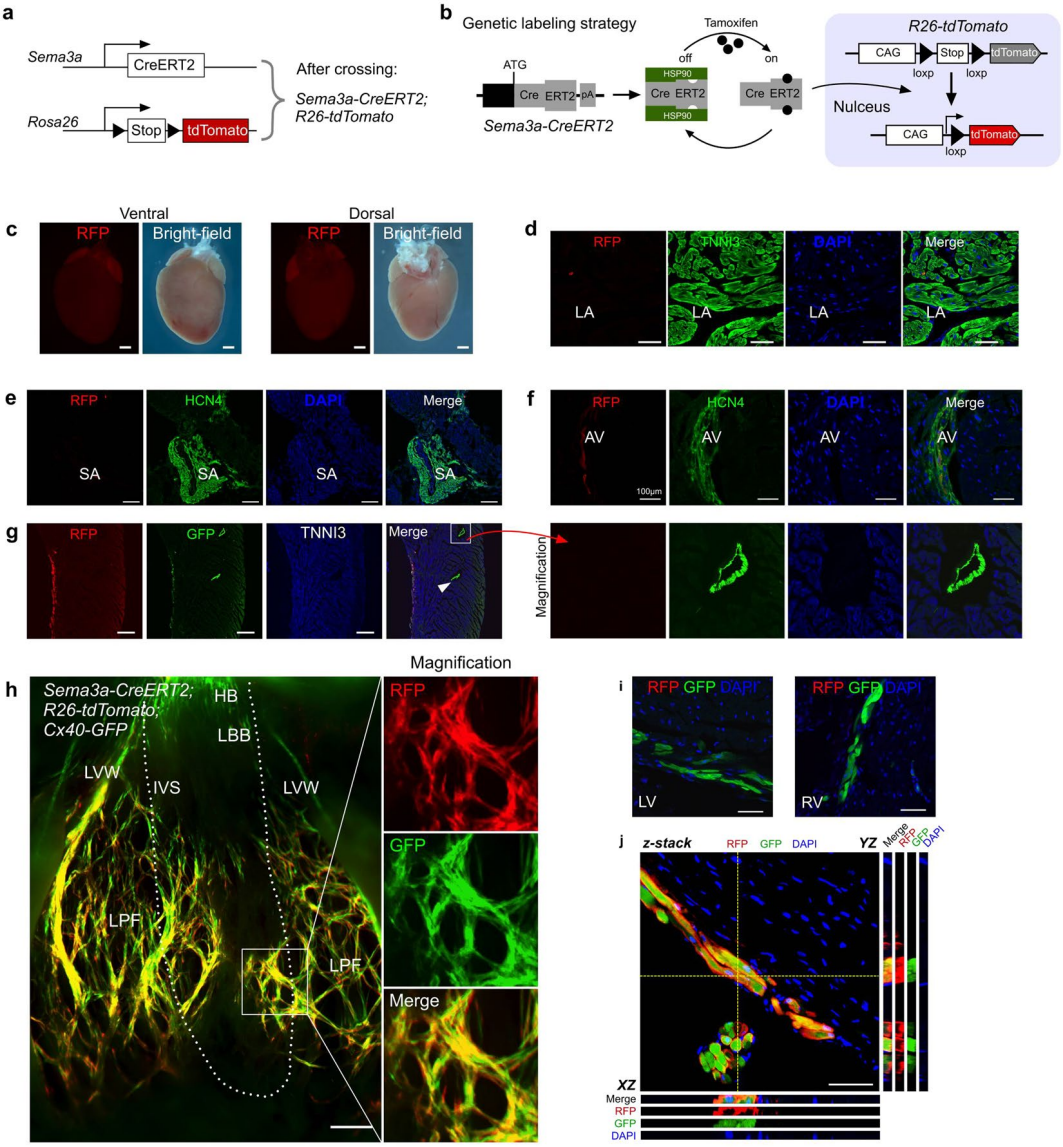
The adult expression map of Sema3a in the heart. (**a**) Schematic showing the crossing of the mice to generate the *Sema3a-CreERT2; R26-tdTomato* mice. (**b**) Genetic labelling of the Sema3a^+^ cells via tamoxifen administration. (**c**) Whole-mount fluorescence and bright-field views of a *Sema3a-CreERT2; R26-tdTomato* mouse heart. (**d**) Immunostaining for RFP and TNNI3 on a *Sema3a-CreERT2; R26-tdTomato* heart section showing the rarity of RFP^+^ cells in the atrium. LA, left atrium. (**e**) No Sema3a^+^ cells were detected in the SA node. (**f**) The expression of Sema3a in the AV node. (**g**) Immunostaining for RFP, GFP and TNNI3 in a *Sema3a-CreERT2; R26-tdTomato; Cx40-GFP* mouse heart section showing that the CX40^+^ coronary artery (arrowhead) was negative for RFP. (**h**) Whole-mount fluorescence view of a *Sema3a-CreERT2; R26-tdTomato; Cx40-GFP* mouse heart. LBB, left bundle branch; LPF, left Purkinje fibre; IVS, interventricular septum; LVW, left ventricular free wall. The dotted line indicates the limits between the IVS and the LVW. (**i**) Immunostaining for RFP and GFP on heart sections of a *Sema3a-CreERT2; R26-tdTomato; Cx40-GFP* mouse. Sema3a was not detected in the LBB or RBB, which were positive for Cx40-GFP. (**j**) Z-stack confocal image showing that Sema3a was expressed in the Purkinje fibres. XZ and YZ indicate the signals from the dotted lines on the Z-stack images in (**j**). Scale bars, 1 mm in (**c**) 500 µm in (**e**,**g**,**h**) and 100 µm in (**d**,**f**,**i**) and (**j**). Each image is representative of 5 individual samples.

According to the ESR staining analysis of the *Sema3a-CreERT2* mice, Sema3a was primarily expressed in the trabecular cardiomyocytes and not in the compact layer. Since Purkinje fibres are restricted in the innermost layer of the ventricular myocardial wall^5^, we next determined whether *Sema3a-CreERT2* could specifically target these specialized cardiomyocytes in the adult mouse heart. We crossed *Sema3a-CreERT2* mice with *R26-tdTomato*^17^ mice to generate *Sema3a-CreERT2; R26-tdTomato* double-positive mice (Fig. 2a). CreERT2 is retained in the cytoplasm, and, only in the presence of tamoxifen^18^, CreERT2 translocates into the nucleus for Cre-loxP recombination (Fig. 2b), which leads to a permanent tdTomato expression in the Sema3a^+^ cells and their descendants^19^. We treated 8-12-week-old mice with tamoxifen and collected the hearts 48 hours after the tamoxifen treatment. We found that very sparse RFP^+^ cells were detected among the atrial cardiomyocytes (Fig. 2c,d). To determine whether Sema3a was expressed in the SA node and the AV node, we co-stained for HCN4 and RFP and found that Sema3a was not expressed in the SA node and was sparsely expressed in the AV node (Fig. 2e,f). With the purpose of detecting whether Sema3a was expressed in the stellate ganglia or cardiac nerves, we co-stained for RFP and tyrosine hydroxylase (TH, which is a marker of the stellate ganglia) and co-stained for RFP and Tuj1 (a marker of cardiac nerves) and found that Sema3a was not expressed in the stellate ganglia or in the cardiac nerves (Supplementary Fig. 4a,b). Because Cx40 has been reported to mark the VCS, we generated *Sema3a CreERT2; R26-tdTomato; Cx40 GFP*^5^ triple-positive mice to test for the expression of Sema3a in the Purkinje fibres. By immunostaining heart sections for RFP and GFP, we found that RFP was co-localized with GFP^+^ cells in the subendocardial layer of the ventricular wall, whereas RFP was not detected in the GFP^+^ coronary arteries in the compact myocardium (Fig. 2g). We next dissected the ventricles for whole-mount fluorescence views of the inside of the *Sema3a-CreERT2; R26-tdTomato; Cx40-GFP* mouse hearts (Fig. 2h and Supplementary Fig. 5). Cx40 exhibited gross expression in the atrium, whereas Sema3a was hardly detected. Cx40 was also expressed in the bundle of His (HB), in the left and right bundle branches, and in the Purkinje fibres, which is consistent with a previous report^5^. Compared with Cx40 (GFP signal), Sema3a (RFP signal) was largely restricted to the Purkinje fibres and was not detected in the bundle of His or the bundle branches (Fig. 2h,i). *Z*-stack confocal microscopy confirmed the expression of Sema3a (RFP signal) in the Cx40^+^ Purkinje fibres that reside in the subendocardial location of the ventricular myocardium (Fig. 2j). The same pattern of Sema3a expression was observed in the right ventricle (Supplementary Fig. 5). These data demonstrated that *Sema3a-CreERT2* specifically targeted the ventricular Purkinje fibres in the adult mouse heart.

### Fate-mapping analysis of Purkinje fibre development with *Sema3a-CreERT2*

**Figure 3:**
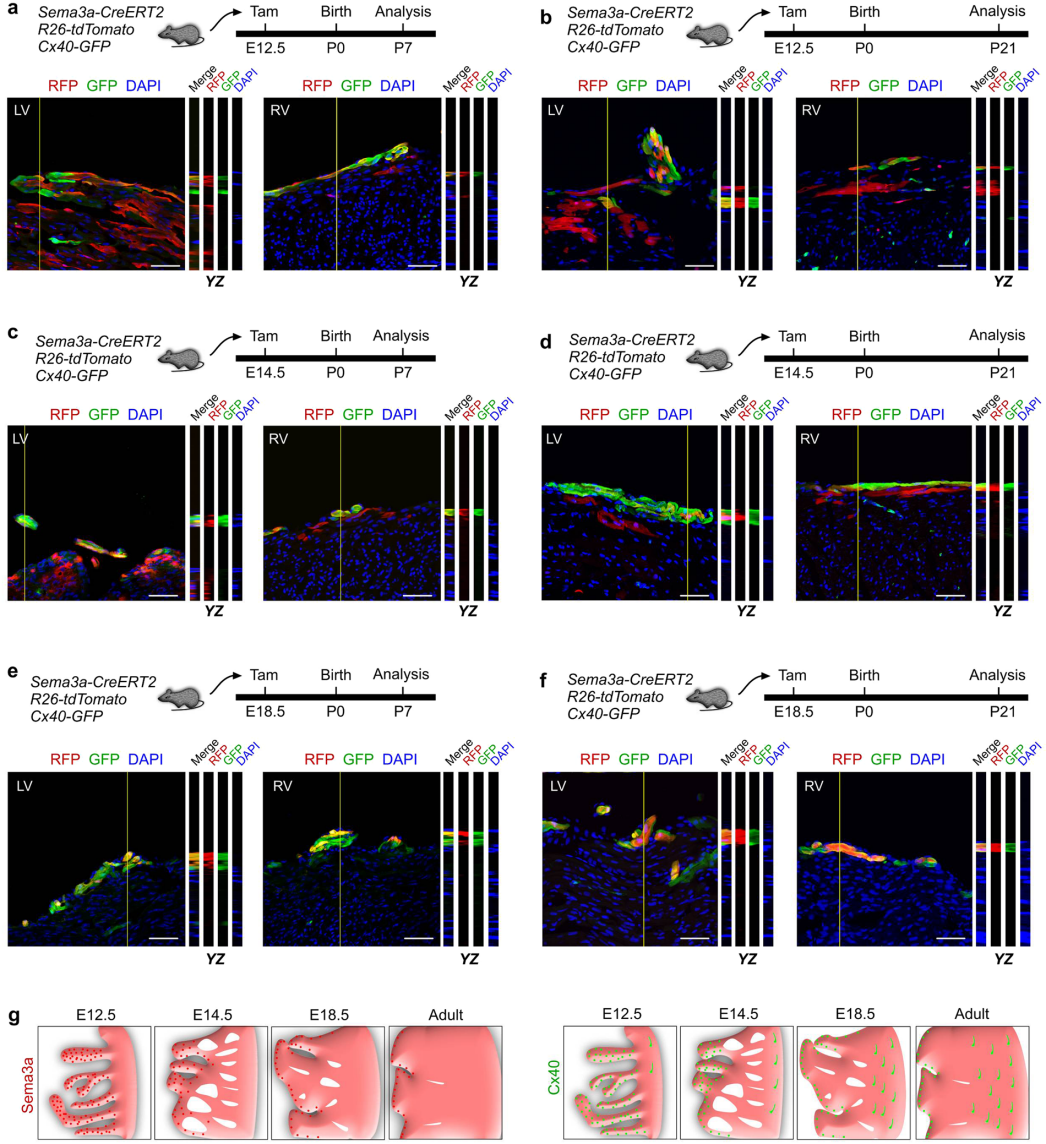
Specialization of Sema3a^+^ cardiomyocytes into the conduction system in the developing heart. (**a**–**f**) Z-stack images of RFP and GFP immunostaining on *Sema3a-CreERT2; R26-tdTomato; Cx40-GFP* heart sections. Tamoxifen was administered at E12.5 (**a**,**b**), E14.5 (**c**,**d**) and E18.5 (**e**,**f**). The hearts were collected at P7 and P21 for each group. YZ indicates signals from the dotted lines on the Z-stack images. Scale bars, 50 µm. Each image is representative of 5 individual samples. (**g**) Schematic figure showing Sema3a^+^ cells (red) and Cx40^+^ cells (green) in the developing and adult heart.

Since Sema3a was expressed in a broader domain during the embryonic stages than in the adults, we asked when Sema3a^+^ cardiomyocytes were specialized to the fate of Purkinje fibres in the developing hearts. We injected tamoxifen at E12.5, E14.5 or E18.5 into *Sema3a-CreERT2; R26-tdTomato; Cx40-GFP* triple-positive mice and collected the hearts at P7 or P21 for analysis. The lineage tracing data revealed that the Sema3a^+^ cells at E12.5 gave rise to both the Cx40^+^ Purkinje fibres (minority) and Cx40^−^ working cardiomyocytes (majority) at P7 and P21 (Fig. 3a,b). The Sema3a^+^ cells at E14.5 were more restricted to the subendocardial layer of the ventricular myocardium and gave rise to the Cx40^+^ Purkinje fibres and Cx40^−^ working cardiomyocytes at P7 and P21 (Fig. 3c,d). The Sema3a^+^ cells at E18.5 gave rise to almost all of the Cx40^+^ Purkinje fibres in the subendocardial surface of the ventricular myocardium at P7 and P21 and gave rise to few if any Cx40^−^ working cardiomyocytes (Fig. 3e,f). These data demonstrated that the fate of Sema3a^+^ cardiomyocytes was specialized to Purkinje fibres in the perinatal stage (e.g., E18.5) when their cell fate was restricted to the ventricular conduction system (Fig. 3g).

### Quantification of the Sema3a^+^ cardiomyocytes

**Figure 4:**
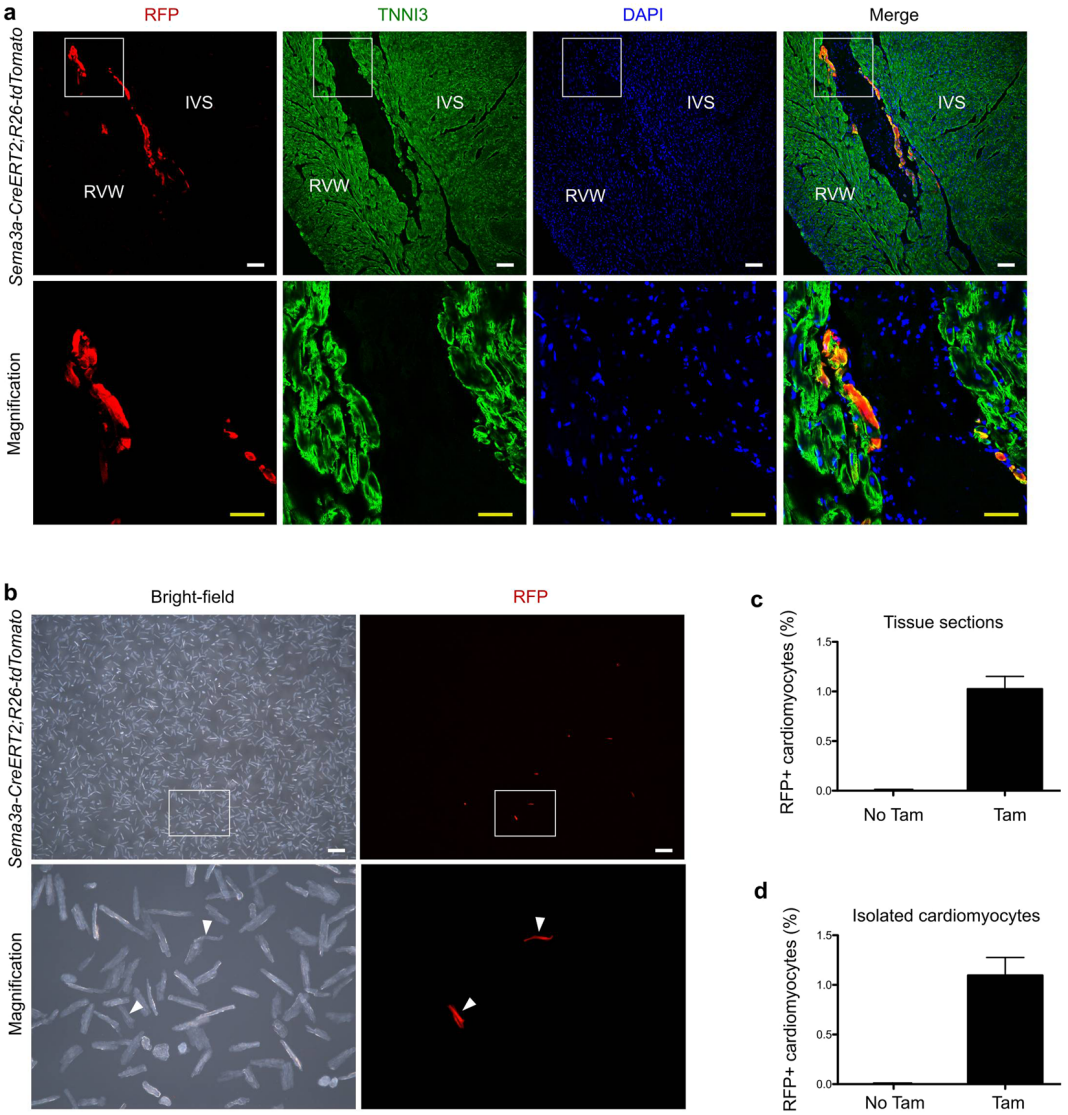
Quantification of the numbers of Sema3a^+^ cardiomyocytes in the adult heart. (**a**) Immunostaining for RFP and TNNI3 on adult heart sections. IVS, interventricular septum; RVW, right ventricular wall. Scale bars: white, 100 µm; yellow, 50 µm. (**b**) Image of isolated cardiomyocytes from the adult *Sema3a CreERT2; R26-tdtomato* heart showed Sema3a^+^ cardiomyocytes. (**c**) Quantification of the percentages of RFP^+^ cardiomyocytes in the heart sections. (**d**) Quantification of the percentages of RFP^+^ cells from among isolated cardiomyocytes. Each image is representative of 5 individual samples.

Finally, we quantified the percentage of cardiomyocytes that were labelled with *Sema3a-CreERT2; R26-tdTomato* in the adult mouse hearts. Tamoxifen was injected into the adult mice, and the hearts were collected for analysis 48 hours after the tamoxifen treatment. Immunostaining of the *Sema3a-CreERT2; R26-tdTomato* hearts for RFP and TNNI3 revealed RFP^+^ cardiomyocytes in the subendocardial surface of the ventricular myocardium (Fig. 4a). Quantification of the percentage of RFP^+^ cardiomyocytes in the heart sections revealed that 1.02 ± 0.13% of the cardiomyocytes were labelled with *Sema3a-CreERT2*, and there were almost no cardiomyocytes that were labelled without the tamoxifen treatment (Fig. 4c). We also isolated cardiomyocytes and placed them in dishes for quantification (Fig. 4b). We found that 1.10 ± 0.18% of the cardiomyocytes were RFP^+^ in the tamoxifen-treated mouse hearts, and very few RFP^+^ cardiomyocytes were present in the mice without the tamoxifen treatment (Fig. 4d). These findings were consistent with the quantification obtained following immunostaining of the heart sections (Fig. 4c).

## Discussion

In this study, we generated a new genetic tool, i.e., *Sema3a-CreERT2*, which specifically targets Purkinje fibres in the adult mouse heart. In contrast, *Hcn4-CreERT2* targets the sinoatrial node, atrioventricular node, the bundle of His, the bundle branches and the Purkinje fibres, and *Cntn2-Cre* is detected in the SA node, AV node, and His-Purkinje system^4^. In contrast to the *Cx40-CreERT2* tool, *Sema3a-CreERT2* does not label coronary arterial endothelial cells or atrial cardiomyocytes. The fate mapping study also revealed that the Sema3a^+^ cells were trabecular cardiomyocytes in the early embryonic stage, and during the perinatal period, the Sema3a^+^ cardiomyocytes specialized into the Purkinje fibre fate. Since our CreERT2 is inducible, and its efficiency is not 100%, the *Sema3a-CreERT2* labelled most, but not all, of the *Cx40-GFP* cells. In the heart, we detected RFP^+^GFP^+^ labelled cells and RFP^−^GFP^+^ unlabelled cells in the P7 and P21 hearts. No significant dilution of RFP labelling efficiency was observed from P7 to P21. It might be possible, although unlikely, that a few Cx40-GFP^+^ cells could be recruited from myocytes at a later stage. Furthermore, tamoxifen treatment lasts 1–2 days for the labelling of cells; we, therefore, cannot formally exclude the possibility that a small number of CreERT2^+^ cells were labelled at a later stage due to incomplete clearance of the tamoxifen from the body. We believe this possibility is unlikely because the majority of tamoxifen is cleared after 1–2 days, and the labelling of the CreERT2^+^ cells afterwards should be minimal. Additionally, the expression domain of CreERT2 (Sema3a) in the myocardium decreases rather than increases during embryonic development (e.g., compare E12.5, E14.5 and E19.5 in Fig. 1c). A broader Cre-loxP recombination domain is, therefore, unlikely even if there is residual tamoxifen remaining after 1–2 days. In conclusion, *Sema3a-CreERT2* is the first genetic mouse tool for the specific targeting of Purkinje fibres and provides a means for the *in vivo* functional study of Purkinje fibres during ventricular conduction system formation and cardiac arrhythmias.

## Materials and Methods

### Animals

All mouse lines used in this study were handled according to the guidelines of the Institutional Animal Care and Use Committee (IACUC) at the Institute for Nutritional Sciences, Shanghai Institute of Biochemistry and Cell Biology, Shanghai Institutes for Biological Science, Chinese Academy of Science (approved protocol number 2015-AN-8). We generated the *Sema3a-CreERT2* knock-in mouse line by knocking a cDNA encoding Cre recombinase fused with a mutant form of the oestrogen receptor hormone-binding domain into the Sema3a locus^20^. Briefly, a 129-bp mouse BAC clone containing the complete mouse Sema3a gene was obtained from the Sanger Institute (UK). The targeting vector was generated using the following cassettes: CreERT2 cDNA, SV40 polyA sequence, and Frt site-flanked PGK-EM7-Neo resistant gene. The neo cassettes were detected by PCR (Fig. 1b). All mice were maintained on a C129/C57BL6/J mixed background. *Sema3a-CreERT2* mice were crossed with *R26-tdTomato* (Ai9)^17^ and *Cx40-GFP*^5^ mice to generate *Sema3a-CreERT2; R26-tdTomato* and *Sema3a-CreERT2; R26-tdTomato; Cx40-GFP* mouse lines for genetic lineage tracing of the Sema3a cells. Tamoxifen (Sigma, T5648) was dissolved in corn oil and introduced by gavage at the indicated times (0.1–0.15 mg/g mouse body weight). To determine the exact timing of the embryonic stages, we checked for a vaginal plug and recorded the result as embryonic day 0.5, then we injected tamoxifen at embryonic days 12.5, 14.5 and 18.5. The day of the mouse birthday was postnatal day 0, and the hearts were collected at postnatal days 7 and 21. To assess the leakiness of the inducible Cre in the absence of tamoxifen, we introduced corn oil by gavage to the *Sema3a-CreERT2; R26-tdTomato* mice in parallel. We designed PCR primers that spanned the genomic DNA and inserted cassette as follows: F1: CCTTTCATTCT GCTTCCTCGG, R1: TGCGAACCTCATCACTCGTTG (to test for the correct target allele of 533 bp); and F1: CCTTTCATTCTGCTTCCTCGG, R2: GCTTTGATGGTTAG CGTTGGG (to test for the wild-type allele of 658 bp); and F2: CGACCACCAAGCGAAAC ATC, R3: AAAGACAGGCAATCCCAGTGAAC (to test for the Neo allele of 790 bp). All mice were bred on a C129/C57BL6/J mixed background. We thank Shanghai Biomodel Organism Co., Ltd. for the mouse generation.

### Immunostaining

Immunostaining was performed according to previous protocols^21^. Briefly, we collected mouse hearts or other organs and washed them in cold PBS several times. Then, the heart tissue was fixed in 4% PFA (Sigma), and the fixation time depended on the size of the organ. In general, the embryonic hearts were fixed for approximately 30 minutes, and the adult hearts were fixed for approximately 1 hour. After fixation, the hearts were dehydrated in 30% sucrose at 4 °C until the tissue was fully penetrated. After soaking the hearts at an optimal cutting temperature (OCT, Sakura) for approximately 1 hour at 4 °C, the tissues were embedded in blocks and frozen in a −80 °C fridge. For the section orientation, the hearts were sectioned in the coronal plane (four chamber orientation). We focused on the trabecular muscle in the embryonic heart and the SA node, AV node, the bundle of His, the bundle branches and the Purkinje fibres in the adult hearts. Cyrosections of 8–10 µm thickness were collected on slides. The tissues were blocked in blocking solution (5% normal donkey serum in PBS with 0.1% Triton X-100) for 30 min at room temperature and subsequently incubated in the first antibodies overnight at 4 °C. After fully washing out the primary antibodies, the tissues were then developed with Alexa fluorescent antibodies (Invitrogen). For weak signals, we used HRP or biotin-conjugated antibodies (Jackson Immunoresearch) with a tyramide signal amplification kit (PerkinElmer). Before mounting the slides, the tissues were counterstained with 4′6-diamidino-2-phenylindole (DAPI, Vector Laboratories). The primary antibodies and dilution ratios were as follows: RFP (Rockland, 600-401-379,1:500); ESR (Abcam, ab27595, prediluted); GFP (Nacalai Tesque, 04404–84, 1:100); GFP (Abcam, ab6662, 1:200); TNNI3 (Abcam, ab56357, 1:100); Cx40 (Alpha Diagnostic, Cx40-A, 1:100); HCN4 (Abcam, ab32675, 1:100); tyrosine hydroxylase (TH, Millipore, AB152, 1:100); and Tuj1 (Covance, MMS-435P, 1:100). The images were acquired with a Zeiss confocal microscope (LSM510), an Olympus confocal microscope (FV1000), and a Zeiss stereo-microscope (AXIO Zoom. V16). To produce the Z-stack confocal images, we scanned 3–4 consecutive XY images on the z-axis with an Olympus confocal microscope (FV1000). Then, the images were analysed with ImageJ (NIH) software. In the stack data, an orthogonal view was used to reveal the merged signals on the XZ and YZ axes. The merged signals and split channels are presented to delineate the signals at a one-cell resolution. Each IHC data is representative of 3–5 individual samples.

### Whole-mount heart imaging

The whole-mount images of the adult heart were collected with a Zeiss stereo-microscope (AXIO Zoom. V16). The hearts were collected from 8–12-week-old mice, and the blood was removed via stimulation with necropsy tools. Then, the heart tissues were washed in cold PBS several times until the solution became limpid. We fixed the hearts in 4% PFA (Sigma) for approximately 1 hour at 4 °C and then washed them with cold PBS 3 times for 5 minutes each time. We removed the atrium with surgical scissors and then split the left and right ventricle walls with a scalpel. To expose the Purkinje fibres, we fixed the hearts in agarose dishes with needles. Then, the images were collected with a Zeiss stereo-microscope.

### Dissociated adult cardiomyocytes

Cardiomyocytes were isolated from the adult hearts as described previously^22^. Briefly, we intraperitoneally injected *Sema3a-CreERT2; R26-tdTomato* mice with 200 µl of heparin (6.25 U/µl). Twenty minutes later, the mice were intraperitoneally anaesthetized with 10% chloral hydrate. The dissected heart was perfused with modified Tyrode’s solution (MTS) for 5 min at a rate of 1 ml/min. The heart was next perfused for approximately 20–30 minutes at 37 °C with an enzyme buffer containing 250 U/ml collagenase type 2 (Worthington, LS004176) and 0.3 U/ml Protease XIV (Sigma, P5147) to dissociate the heart tissues. Micro-dissecting forceps were used to mince the ventricles into small pieces for dissociation and filtering with a 100-µm strainer. The isolated cells were centrifuged at 20× *g* for 3 minutes at 4 °C to allow for the collection of most of the cardiomyocytes.

### Cardiomyocyte quantification

Cardiomyocytes were isolated from *Sema3a-CreERT2; R26-tdTomato* mice hearts within 48 hours of the injection of tamoxifen. All cardiomyocytes were collected in cell culture dishes, and the RFP^+^ cardiomyocytes were quantified under a Zeiss stereo-microscope (AXIO Zoom. V16). The total number of RFP^+^ cells from 5 hearts was divided by the total number of cardiomyocytes examined to obtain the percentage of RFP^+^ cardiomyocytes.

### Statistical analysis

All data in this study were determined from 3–5 independent experiments and are presented as the mean values ± the s.e.m. All mice in this study were randomly assigned to the different experimental groups.

## Electronic supplementary material

Supplementary information
