# Supplementary material for: Genetic targeting of Purkinje fibres by Sema3a-CreERT2
Source: Sci Rep. 2018 Feb 5;8:2382. doi: 10.1038/s41598-018-20829-9 (PMC5799187; doi:10.1038/s41598-018-20829-9)
Supplement: Supplementary file 1 — Supplementary information [file 41598_2018_20829_MOESM1_ESM.pdf]

Genetic targeting of Purkinje fibres by *Sema3a-CreERT2*

Yan Li[1,2], Xueying Tian[1,2], Huan Zhao[1,2], Lingjuan He[1,2], Shaohua Zhang[1,2], Xiuzhen Huang[1,2], Hui Zhang[1,2], Lucile Miquerol[3], Bin Zhou[1,2,4,5]

[1]The State Key Laboratory of Cell Biology, CAS Center for Excellence in Molecular Cell Science, Shanghai Institute of Biochemistry and Cell Biology, Chinese Academy of Sciences, University of Chinese Academy of Sciences, Shanghai, 200031, China.

[2]Key Laboratory of Nutrition and Metabolism, Institute for Nutritional Sciences, Shanghai Institutes for Biological Sciences, Chinese Academy of Sciences, University of Chinese Academy of Sciences, Shanghai, 200031, China.

[3]Aix Marseille University, CNRS, IBDM UMR 7288, 13288, Marseille, France.

[4]Key Laboratory of Regenerative Medicine of the Ministry of Education, Institute of Aging and Regenerative Medicine, Jinan University, Guangzhou, 510632, China.

[5]School of Life Science and Technology, ShanghaiTech University, Shanghai, 201210, China.

Correspondence should be addressed to B.Z. (zhoubin@sibs.ac.cn)

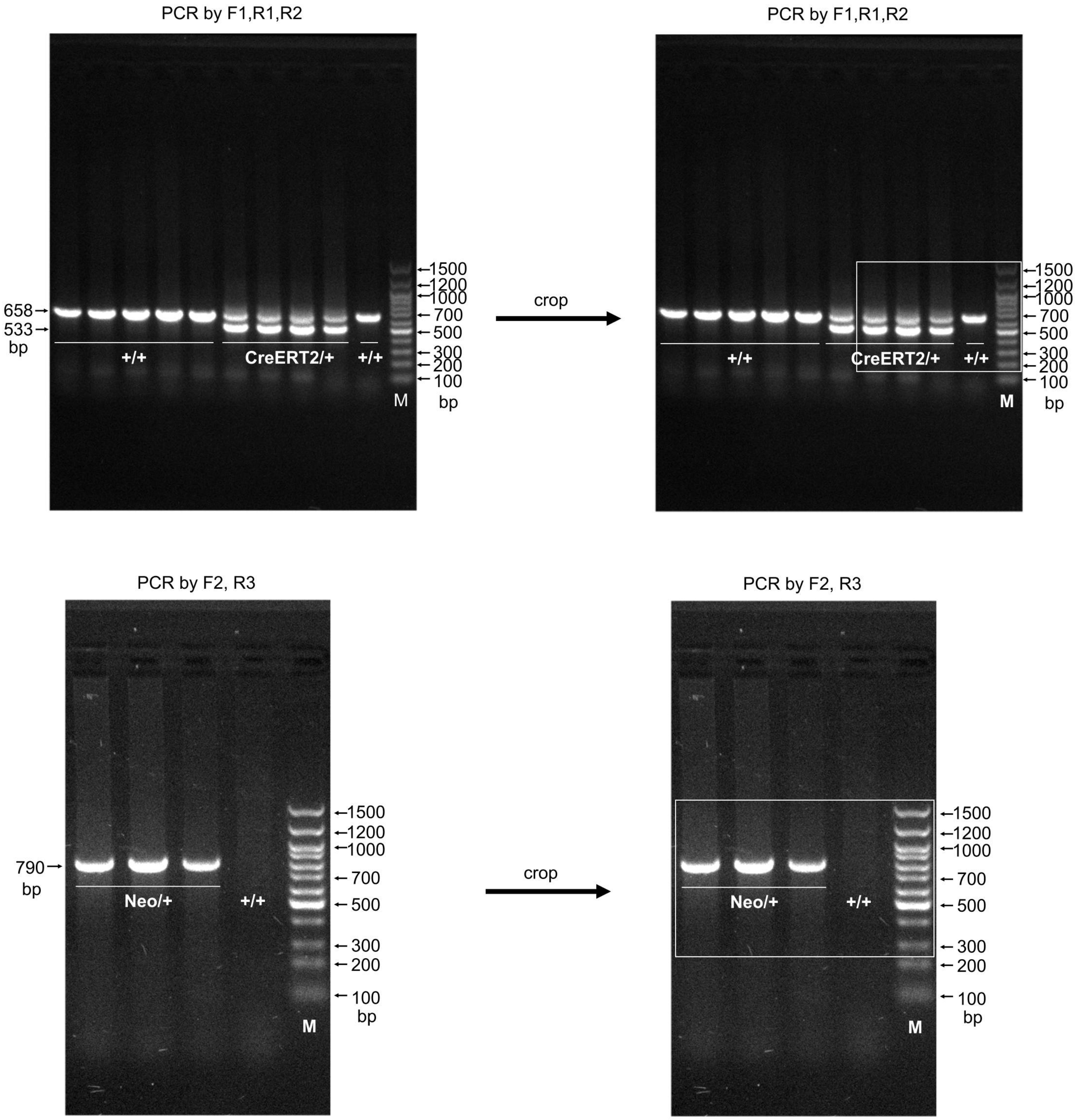

Supplementary Figure 1. The un-cropped PCR gel pictures of *Sema3a-CreERT2* allele. The band size of *Sema3a-CreERT2* mouse was shown on the left. M, molecular marker. The band size of molecular marker was shown on the right. The right panel showed the cropped place of the left panel.

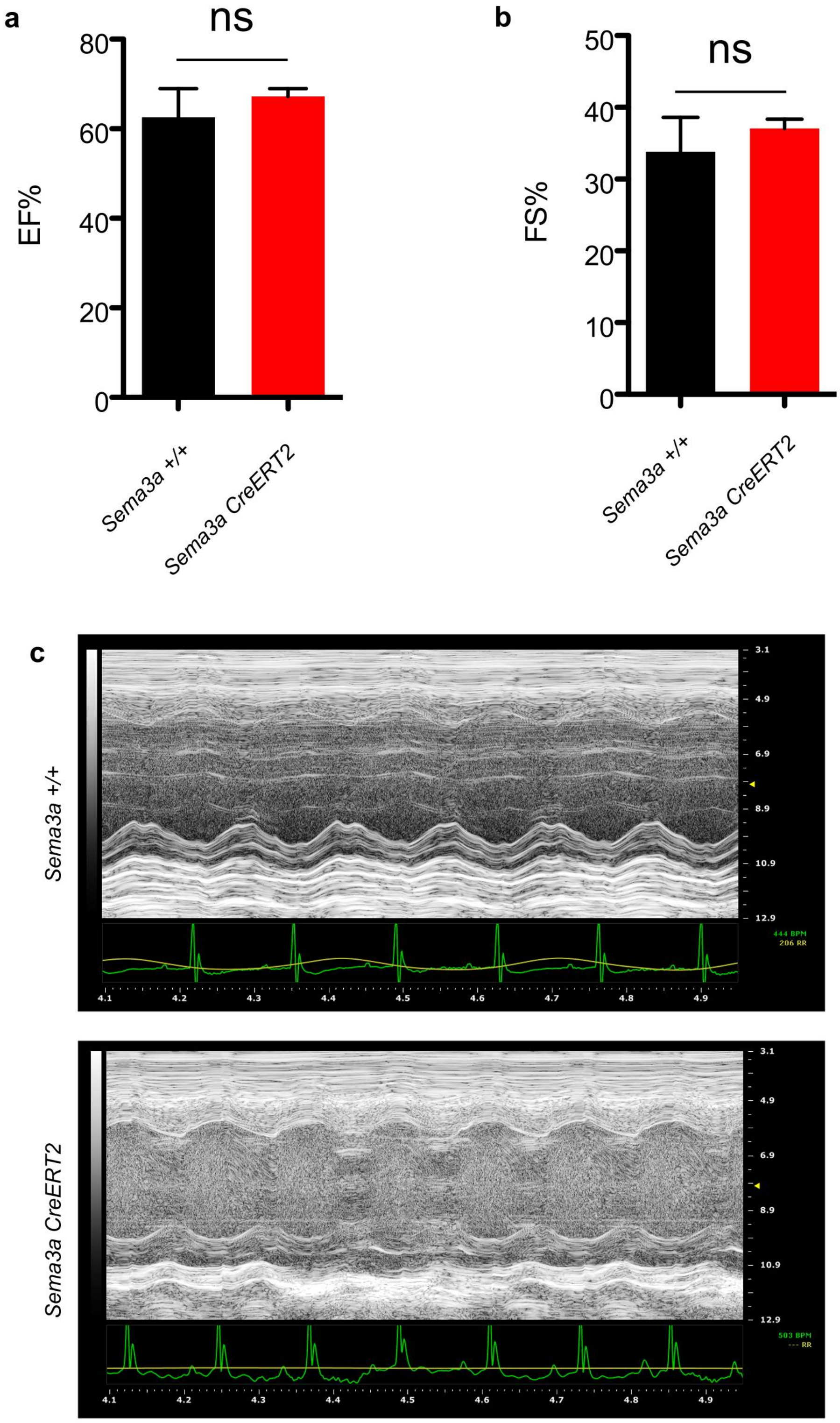

Supplementary Figure 2. The function analysis of *Sema3a CreERT2* mouse line. (a) The ejection fraction of *Sema3a CreERT2* and *Sema3a* $^{+/+}$ mice. **(b)** The fractional shortening of *Sema3a CreERT2* and *Sema3a* $^{+/+}$ mice. ns, no significance. **(c)** The ECG and echocardiography analysis of *Sema3a CreERT2* and *Sema3a* $^{+/+}$ mice. Each experiment was representative of 3 individual samples. Each emperiment was representative of 3 individual samples**.**

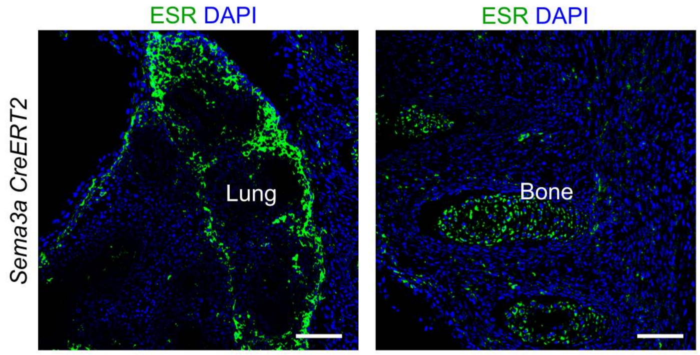

Supplementary Figure 3. The expression map of Sema3a in embryonic lung and bone. ESR staining illustrated that Sema3a was highly expressed in embryonic lung and bone. Scale bars, 100μm.

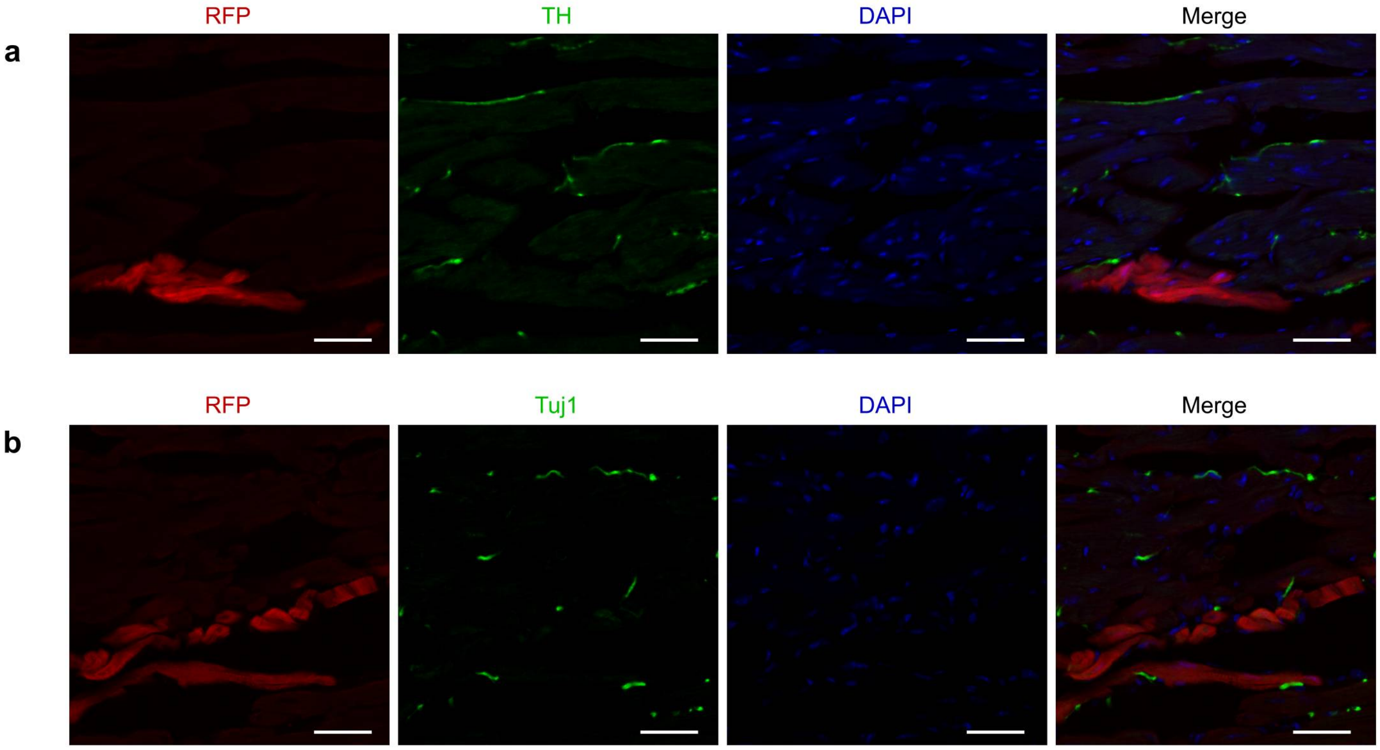

Supplementary Figure 4. Sema3a was not expressed in stellate ganglia or cardiac nerves. (a) Stellate ganglia on *Sema3a CreERT2; R26-tdTomato* heart section was observed by TH immunostaining, Sema3a (RFP signals) was not overlay with TH. **(b)** Co-staining RFP and Tuj1 on *Sema3a CreERT2; R26-tdTomato* heart sections indicated that Sema3a was not expressed in cardiac nerves. Scale bars,100μm.

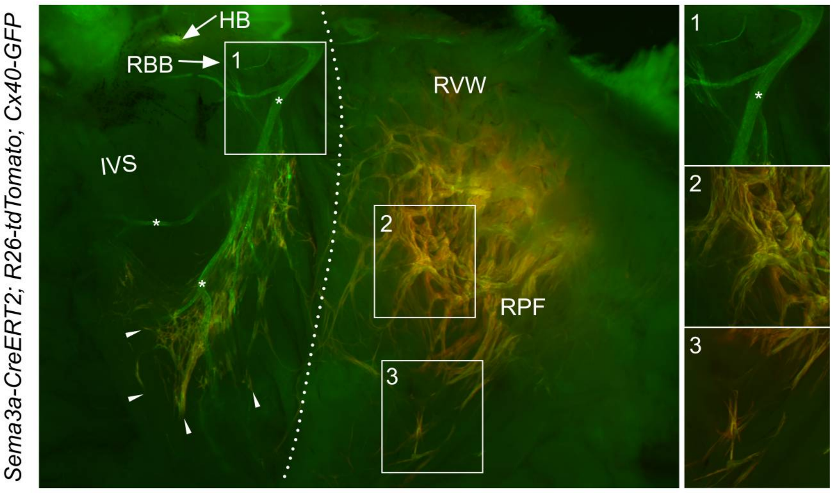

Supplementary Figure 5. The expression map of Sema3a in the right ventricular. Whole-mount fluorescence view of *Sema3a-CreERT2; R26-tdTomato; Cx40-GFP* mouse heart. Magnification of the white box was shown on the right. The whole RVW was exposed on the right. The dotted line indicated the limits between the IVS and the RVW.Arrowheads indicated connecting fiber which had been cut. Asterisk indicated septal artery. HB, His bundle; RBB, right bundle branch; IVS, interventricular septum; RVW, right ventricular free wall; RPF, right Purkinje fiber. Scale bars,1mm.
